# Brain and blood biomarkers of tauopathy and neuronal injury in humans and rats with neurobehavioral syndromes following blast exposure

**DOI:** 10.1038/s41380-020-0674-z

**Published:** 2020-02-25

**Authors:** Dara L. Dickstein, Rita De Gasperi, Miguel A. Gama Sosa, Georgina Perez-Garcia, Jennifer A. Short, Heidi Sosa, Gissel M. Perez, Anna E. Tschiffely, Kristen Dams-O’Connor, Mariel Y. Pullman, Karin Knesaurek, Andrew Knutsen, Dzung L. Pham, Lale Soleimani, Barry D. Jordan, Wayne A. Gordon, Bradley N. Delman, Gleb Shumyatsky, Pashtun-Poh Shahim, Steven T. DeKosky, James R. Stone, Elaine Peskind, Kaj Blennow, Henrik Zetterberg, Steven A. Chance, Mario Torso, Lale Kostakoglu, Mary Sano, Patrick R. Hof, Stephen T. Ahlers, Sam Gandy, Gregory A. Elder

**Affiliations:** 1 Department of Pathology, Uniformed Services University of Health Sciences, 4301 Jones Bridge Rd, Bethesda, MD 20814 USA; 2 Center for Neuroscience and Regenerative Medicine, Bethesda, MD 20814 USA; 3 Research and Development Service, James J. Peters Department of Veterans Affairs Medical Center, 130 West Kingsbridge Road, Bronx, NY 10468 USA; 4 Department of Psychiatry, Icahn School of Medicine at Mount Sinai, One Gustave Levy Place, New York, NY 10029 USA; 5 Department of Neurology, Icahn School of Medicine at Mount Sinai, One Gustave Levy Place, New York, NY 10029 USA; 6 Department of Neurotrauma, Naval Medical Research Center, 503 Robert Grant Avenue, Silver Spring, MD 20910 USA; 7 Department of Rehabilitation and Physical Medicine, Icahn School of Medicine at Mount Sinai, One Gustave Levy Place, New York, NY 10029 USA; 8 Department of Molecular and Interventional Radiology, Icahn School of Medicine at Mount Sinai, One Gustave L. Levy Place, New York, NY 10029 USA; 9 Burke Rehabilitation and Research, 785 Mamaroneck Avenue, White Plains, NY 10605 USA; 10 Department of Genetics, Rutgers University, Piscataway, NJ 08854 USA; 11 National Institute of Neurological Disorders and Stroke, NIH Neurological Institute, PO Box 5081, Bethesda, MD 20824 USA; 12 Department of Neurology, University of Florida, Gainesville, FL 32611 USA; 13 Department of Radiology and Medical Imaging, University of Virginia, Charlottesville, VI 2903 USA; 14 VA Northwest Network Mental Illness Research, Education, and Clinical Center, Seattle, WA 98195 USA; 15 Department of Psychiatry and Behavioral Sciences, University of Washington, Seattle, WA 98195 USA; 16 Institute of Neuroscience and Physiology, Department of Psychiatry and Neurochemistry, The Sahlgrenska Academy at the University of Gothenburg, S-431 80 Mölndal, Sweden; 17 Clinical Neurochemistry Laboratory, Sahlgrenska University Hospital, S-431 80 Mölndal, Sweden; 18 UK Dementia Research Institute at UCL, London, WC1E 6BT UK; 19 Department of Neurodegenerative Disease, UCL Institute of Neurology, Queen Square, London, WC1N 3BG UK; 20 Nuffield Department of Clinical Neurosciences, University of Oxford, Neuropathology, West Wing, Level 1, Oxford, OX3 9DU UK; 21 Mount Sinai Alzheimer’s Disease Research Center and Ronald M. Loeb Center for Alzheimer’s Disease, Icahn School of Medicine at Mount Sinai, New York, NY 10029 USA; 22 Nash Family Department of Neuroscience and Friedman Brain Institute, Icahn School of Medicine at Mount Sinai, One Gustave Levy, New York, NY 10029 USA; 23 Department of Geriatrics and Palliative Care, Icahn School of Medicine at Mount Sinai, New York, NY 10029 USA; 24 NFL Neurological Care Center, Icahn School of Medicine at Mount Sinai, New York, NY 10029 USA; 25 Neurology Service, James J. Peters Department of Veterans Affairs Medical Center, 130 West Kingsbridge Road, Bronx, NY 10468 USA; 26 South Australian Health and Research Medical Institute, Adelaide, 5000 South Australia

**Keywords:** Neuroscience, Biomarkers

## Abstract

Traumatic brain injury (TBI) is a risk factor for the later development of neurodegenerative diseases that may have various underlying pathologies. Chronic traumatic encephalopathy (CTE) in particular is associated with repetitive mild TBI (mTBI) and is characterized pathologically by aggregation of hyperphosphorylated tau into neurofibrillary tangles (NFTs). CTE may be suspected when behavior, cognition, and/or memory deteriorate following repetitive mTBI. Exposure to blast overpressure from improvised explosive devices (IEDs) has been implicated as a potential antecedent for CTE amongst Iraq and Afghanistan Warfighters. In this study, we identified biomarker signatures in rats exposed to repetitive low-level blast that develop chronic anxiety-related traits and in human veterans exposed to IED blasts in theater with behavioral, cognitive, and/or memory complaints. Rats exposed to repetitive low-level blasts accumulated abnormal hyperphosphorylated tau in neuronal perikarya and perivascular astroglial processes. Using positron emission tomography (PET) and the [^18^F]AV1451 (flortaucipir) tau ligand, we found that five of 10 veterans exhibited excessive retention of [^18^F]AV1451 at the white/gray matter junction in frontal, parietal, and temporal brain regions, a typical localization of CTE tauopathy. We also observed elevated levels of neurofilament light (NfL) chain protein in the plasma of veterans displaying excess [^18^F]AV1451 retention. These findings suggest an association linking blast injury, tauopathy, and neuronal injury. Further study is required to determine whether clinical, neuroimaging, and/or fluid biomarker signatures can improve the diagnosis of long-term neuropsychiatric sequelae of mTBI.

## Introduction

Concern exists over the role of traumatic brain injury (TBI) in the chronic cognitive and behavioral symptoms that may develop during or after military service [1]. It is estimated that approximately 10–20% of veterans returning from the conflicts in Iraq and Afghanistan sustained mild TBI (mTBI) resulting from the shockwaves emitted from blast exposures especially those related to improvised explosive devices (IEDs) [2–4]. The true prevalence of mTBI may be even higher, given that many blast-related injuries go undocumented [5]. Symptoms following mTBI frequently resolve in days to months following injury; however, in a subset of patients, symptoms persist and evolve into a chronic syndrome [6]. It is estimated that 7.5–40% of veterans who experience blast-related mTBI endorse three or more post-concussive symptoms at 3 months following the incident [7–10]. In addition to static symptoms that persist, new symptoms may also appear months to years following TBI, including those associated with delayed neurodegeneration and dementia [1, 11]. Estimates as to the size of this population vary, but one study of U.S. military service personnel who experienced an mTBI in Iraq or Afghanistan found that—while many improved—about 20% reported the appearance of new symptomatology, such as posttraumatic-stress disorder (PTSD) within 5 years after injury [12, 13]. Another study of 188,764 older veterans (mean age of 68 years) revealed that a history of any TBI was associated with a 60% increase in the risk of developing dementia over a 9-year follow up [14–16].

Symptoms of post-traumatic neurodegeneration are variable but often include memory loss along with alterations in mood or personality, as well as poor impulse control and aggression. The pathology underlying these symptoms may represent Alzheimer’s disease (AD)-type dementia or another clinical entity known as chronic traumatic encephalopathy (CTE). CTE was first recognized in boxers and initially designated as “punch drunk syndrome” and later designated “dementia pugilistica” [17]. Clinical features of CTE include a progressive cognitive, neurological, and behavioral syndrome; however, while CTE can be suspected during life, CTE can only be confirmed postmortem. CTE is characterized by aggregates of phosphorylated tau deposited as neurofibrillary tangles (NFTs) inside neurons and within astrocytes localized near vascular structures especially in the depths of the sulci in the superficial cortical layers [18]. Other features include variable frequency and severity of axonal damage and neuronal loss, astrogliosis, and deposition of amyloid-β peptide (Aβ) and/or TAR DNA-binding protein 43 (TDP43) [19].

There have been a few reports of pathologically confirmed cases of CTE in the military veteran population [20–22]. In addition to classical CTE pathology, Shively et al. [23]. describe a novel astroglial pathology, wherein scar-like changes involved the subpial glial plate, penetrating cortical blood vessels, white/gray matter junction, and structures lining the ventricles. In another postmortem study, axonal pathology without demonstrable tauopathy was also observed [24]. The prevalence of these pathological subtypes in veterans with chronic cognitive/behavioral syndromes following blast or other types of TBI remains to be determined. Interestingly, other researchers have described an increase in plasma tau concentrations in recently deployed veterans with a history of TBI, and the levels of plasma tau were reported to correlate with the severity of symptoms [25].

Based on the range of findings from multiple postmortem analyses, it is important to specify the pathological consequences of blast exposure and to determine whether we can detect some or all of these pathologies in living individuals. In the present study, we utilized our well-established and characterized battlefield-relevant rat model of blast-induced mTBI with chronic anxiety-related traits [26–32] to perform a clinicopathological study of tauopathy side by side with a clinical biomarker study of its human counterpart. In order to detect neuropathological alterations in veterans that have been exposed to blast, we performed positron emission tomography (PET) brain imaging using the amyloid ligand [^18^F]AV45 and the tau ligand [^18^F]AV1451. As an additional biomarker, we measured blood levels of neurofilament protein light chain (NfL), since elevated levels of NfL have been reported in human patients suffering from a variety of brain injuries including mTBI and neurodegenerative diseases [33]. The results here indicate that NfL might be an informative parameter to include in a biomarker panel aimed at enabling the diagnosis of CTE during life.

## Materials and methods

### Animals

Adult male Long Evans hooded rats (250–350 g; 10 weeks of age; Charles River Laboratories International, Wilmington, MA, USA) were used. All studies involving animals were reviewed and approved by the Institutional Animal Care and Use Committees of the Walter Reed Army Institute of Research (WRAIR)/Naval Medical Research Center and the James J. Peters VA Medical Center. Studies were conducted in compliance with the Public Health Service policy on the humane care and use of laboratory animals, the NIH Guide for the Care and Use of Laboratory Animals, and all applicable Federal regulations governing the protection of animals in research.

### Blast overpressure exposure

Rats were exposed to overpressure injury using the WRAIR shock tube, which simulates the effects of air blast exposure under experimental conditions. The shock tube has a 12-inch circular diameter and is a 19.5 ft. long steel tube divided into a 2.5 ft. compression chamber that is separated from a 17 ft. expansion chamber. The compression and expansion chambers are separated by polyethylene Mylar ^TM^ sheets (Du Pont Co., Wilmington, DE, USA) that control the peak pressure generated. The peak pressure at the end of the expansion chamber was determined by piezoresistive gauges specifically designed for pressure-time (impulse) measurements (Model 102M152, PCB, Piezotronics, Inc., Depew, NY, USA). This apparatus has been used in multiple prior studies to deliver blast overpressure injury to rats [26–31, 34].

Individual rats were anesthetized using an isoflurane gas anesthesia system consisting of a vaporizer, gas lines and valves and an activated charcoal scavenging system adapted for use with rodents. Rats were placed into a polycarbonate induction chamber, which was closed and immediately flushed with 5% isoflurane in air mixture for 2 min. Rats were placed into a cone shaped plastic restraint device and then placed in the shock tube. Movement was further restricted during the blast exposure using 1.5 cm diameter flattened rubber tourniquet tubing. Three tourniquets were spaced evenly to secure the head region, the upper torso and lower torso while the animal was in the plastic restraint cone. The end of each tubing was threaded through a toggle and run outside of the exposure cage, where it was tied to firmly affix the animal and prevent movement during the blast overpressure exposure without restricting breathing. Rats were randomly assigned to sham or blast conditions and were placed in the shock tube lying prone with the plane representing a line from the tail to the nose of the body in line with the longitudinal axis of the shock tube with the head placed more upstream. Further details of the physical characteristics of the blast wave are described in Ahlers et al. [34]. Blast exposed animals received 74.5 kPa (equivalent to 10.8 psi, duration 4.8 ms, impulse 175.8 kPa ms) exposures administered one exposure per day for 3 consecutive days. Sham exposed animals were treated identically including receiving anesthesia and being placed in the blast tube but did not receive a blast exposure. Within 10 days after the last blast or sham exposure animals were transported in a climate-controlled van from the WRAIR to the James J. Peters VA Medical Center (Bronx, NY, USA). Animals left in the morning from the WRAIR and arrived in the afternoon of the same day at the James J. Peters VA Medical Center, where all other procedures were performed.

### Western blotting

Animals were euthanized by CO_2_ narcosis between 6 weeks and 12 months post-blast and the brains quickly removed and regionally dissected [35]. Preparations of extracts, Western blot analysis and quantification were performed as described [26] (see Supplementary Methods). The primary antibodies utilized are indicated in Supplementary Table 1. Phosphorylated tau (p-tau) levels were determined by adding the two major bands seen in Western blot and normalizing to total tau. For total tau quantitation the sum of the major and minor bands seen in Western blot was used. Total tau levels were normalized to glyceraldehyde 3-phosphate dehydrogenase (GAPDH). All data were normalized relative to control samples. At 6 weeks post-blast exposure four controls and five blast-exposed animals were analyzed while at 10 months post-blast exposure, five control and five blast-exposed animals were analyzed.

### Histology and immunohistochemistry

Rats utilized for histological studies were perfused with 4% paraformaldehyde in PBS, postfixed in 4% paraformaldehyde and cut on a Vibratome (Leica, Wetzlar, Germany) into 50 μm-thick coronal sections as described [27]. Immunohistochemical staining was performed as previously described [27] using the antibodies indicated in Supplementary Table 1. Five blast-exposed and five control animals were analyzed at 6 weeks post blast exposure; seven blast-exposed and seven controls were analyzed between 4.5 and 10 months after blast exposure. Stained sections were imaged with a Zeiss 700 confocal microscope (Zeiss, Thornwood, USA) and the images processed with Adobe Photoshop CC (Adobe Systems, San Jose, CA, USA).

### Human subjects

Subjects were recruited at the Icahn School of Medicine at Mount Sinai (ISMMS) and informed written consent was obtained from all participants. All procedures were approved by the ISMMS Institutional Review Board. A total of 17 subjects (10 veterans, mean age 41.20 ± 9.42, and seven healthy comparison subjects, mean age 47.33 ± 7.06; all male, all right handed) are reported here and were seen at ISMMS from various sources including one from the VA Northwest Network Mental Illness Research, Education, and Clinical Center, Seattle WA (Dr. Elaine Peskind), seven from the recruitment core at the Center for Neuroscience and Regenerative Medicine (CNRM) at the Uniformed Services University of Health Sciences in Bethesda, MD, and two through clinicaltrials.gov. Age comparable, healthy subjects with no history of head injury, behavioral, or cognitive complaints were selected through local advertisements and social media. All included veterans reported histories of repetitive blast exposure and met VA/Department of Defense/American College of Rehabilitation Medicine criteria for mTBI. mTBI histories were confirmed by study investigators based on retrospectively recalled acute CNS symptoms at the time of exposure. All veterans also reported behavioral and cognitive complaints (Table 1). Diagnosis of PTSD was based on review of veteran’s VA and other medical records and/or reported to the study doctor by the subject and informant during the doctor’s visit portion of the study. Exclusion criteria for both veteran and comparison group participants included any significant medical illness, neurological disease or psychiatric disorder (other than depression or PTSD for the veterans), moderate-to-severe TBI, systemic cancer, history of substance use/abuse and current alcohol or other drug dependence within the past year, reported history of concussion requiring hospitalization, education level <10 years, or the presence of any MRI-incompatible prostheses or ferromagnetic metal. Participants completed a comprehensive neuropsychological battery (Table 2) and underwent a standard medical exam performed by a clinician.

**Table 1:** Subject clinical symptoms, TBI history, CDA, plasma NfL, and imaging results.

Subject	Age	TBI history	Last TBI	Date seen	CDA	NfL (pg/ml)	[^18^F]AV1451	[^18^F]AV45 SUVr	MRI	Symptoms^a^
Control 1	42	0	N/A	2017	0	N/A	Negative	0.97	Normal	None
Control 2	45	0	N/A	2016	0	N/A	Negative	1.01	Normal	None
Control 3	53	0	N/A	2015	0	4.6	Negative	0.89	Scattered periventricular and subcortical T2/FLAIR hyperintensities, most in the right centrum semiovale	None
Control 4	60	0	N/A	2017	0	21.7	Negative	0.99	Normal	None
Control 5	49	0	N/A	2016	1	9.6	Negative	0.87	Normal	None
Control 6	38	0	N/A	2017	0	6.8	Negative	0.95	Normal	None
Control 7	57	0	N/A	2016	0	N/A	Negative	0.94	Minimal scattered T2/FLAIR hyperintense foci within bilateral periventricular and subcortical WM	None
Veteran 1	32	4 IEDs, 2 TBI, 2 LOC	2008	2017	1	7.6	Positive	0.89	Few scattered T2/FLAIR hyperintense foci in the WM, partially empty sella	Dx TBI and PTSD, Depression, anxiety, suicidality, poor memory and concentration, headaches, sleep disturbance, dizziness, resting tremors
Veteran 2	35	3 IEDs, 1TBI, No LOC	2006	2017	1	13.6	Positive	0.92	Normal	Dx of PTSD, headaches, blurred vision, sleep disturbances, fatigue, depression, irritability, poor memory and concentration, restlessness
Veteran 3	32	~5 IEDs, 5 TBIs, 1 LOC	2010	2017	1	9.9	Negative	0.87	Multiple small foci of T2 hyperintensity within the periventricular and subcortical WM	Dx PTSD, headaches, depression, anxiety, sleep disturbances, irritability, poor memory
Veteran 4	55	1 IED, 3 TBI, 1 LOC	2010	2017	0	25.9	Positive	0.93	Normal	Headaches, dizziness, poor memory and concentration, aggression, light sensitivity
Veteran 5	42	~50 TBIs, 5 LOC	1997	2017	1	12.8	Positive	0.96	Normal	Dx PTSD and TBI, headaches, depression, irritability, sleep disturbances, fatigue, restlessness, light sensitivity, poor memory and concentration
Veteran 6	41	1 IED, 11 TBIs, no LOC	2004	2017	0	3.6	Negative	0.88	Osseous lesion located on the roof of the left orbit	Poor memory and concentration, light sensitivity, depression, sleep disturbance, irritability, fatigue, dizziness, restlessness
Veteran 7	58	~10 IEDs, 15 TBI, No LOC	2002	2017	0	9.4	Negative	0.86	Non-specific scattered T2/FLAIR hyperintense foci within the periventricular and deep cortical WM regions	Headaches, dizziness, irritability, poor memory and concentration, fatigue, light sensitivity, blurred vision, restlessness
Veteran 8	47	4 IEDs, ~21 TBI, 4 LOC	2008	2017	0	4.4	Negative	0.91	Prominent rightward nasal septal deviation. There are multifocal T2/FLAIR hyperintense foci in the subcortical, deep and periventricular WM regions	Dx PTSD, Poor memory and concentration, apathy, anxiety, depression, restlessness
Veteran 9	37	~40 IEDs, 2 TBI	2004	2017	1	19	Positive	0.93	Normal	Irritability, Anxiety, depression
Veteran 10	33	Many IEDs, 3 TBI, 2 LOC	2009	2017	N/A	1.7	Negative	0.94	Normal	Dx PTSD, headaches, dizziness, fatigue, sleep disturbances, depression, irritability, poor memory and concentration, suicidality

Three controls did not have NfL measurements performed. One veteran did not have CDA analysis performed. [^18^F]AV1451 PET positive subjects (qualitative reads by radiologist).

^a^The control group responded to advertisements or requests through social media for subjects without cognitive complaints and no history of TBI. Screening of control subjects included an interview in which subjects were asked about common neurological complaints. Symptoms from the veteran group were ascertained by medical record review and by subject or informant complaint.

**Table 2:** Behavioral and neuropsychological performance.

Test	Veteran (*n* = 10)	Control (*n* = 7)	*P*-value
AUDIT	5.22 ± 5.25	4.29 ± 6.01	0.549
BDI-II	17.67 ± 7.76	5.43 ± 4.31	0.004
*Cognitive function*			
MoCA	23.33 ± 2.55	26.86 ± 1.86	0.022
WRAT (IQ)^**a**^	100 ± 7.51	118.5 ± 11.44	0.026
*WAIS-IV: (ss)*			
Digit span forward	9.10 ± 2.38	11.14 ± 3.76	0.197
Digit span backwards	10.10 ± 2.85	10.86 ± 3.58	0.806
Symbol search	8.78 ± 3.19	9.57 ± 2.99	0.961
Digit symbol coding	7.30 ± 2.50	11.86 ± 2.25	0.003
Stroop color-word (*t*-score)	43.22 ± 7.71	53.71 ± 14.23	0.056
Grooved Peg Board (*t*-score)	38.00 ± 13.96	49.43 ± 9.16	0.04
Trail making test Part A (t-score)	49.45 ± 15.81	51.57 ± 6.74	0.812
Trail making test Part B (*t*-score)	41.78 ± 18.05	53.70 ± 11.08	0.229
*CVLT (t-score)*			
Total trails 1–5	48.30 ± 9.60	58.29 ± 13.67	0.106
SDFR	49.00 ± 10.75	51.43 ± 8.99	0.692
LDFR	47.50 ± 14.56	53.57 ± 8.52	0.519
*BVMT (t-score)*			
Total recall	44.56 ± 11.73	48.43 ± 8.50	0.596
Delayed recall	47.56 ± 14.35	56.29 ± 7.82	0.204

*AUDIT* alcohol use disorders identification test, *CVLT* California verbal learning Test, *SDFR* short delay free recall, *LDFR* long delay free recall, *WRAT* wide range achievement test, *WAIS-IV* Weschler Adult Intelligence Scale, *BVMT* brief visuospatial memory test.

^a^Two veterans and one control did not perform this task.

### MRI acquisition and analysis

All subjects underwent magnetic resonance imaging (MRI) for structural analysis as well as for anatomic delineation of regions of interest (ROIs) on PET images after PET-MRI co-registration. The MRI T1-weighted images were obtained on a 3-Tesla Siemens Biograph MR system (Siemens Healthcare, Erlangen, Germany) (see Supplementary Methods). MRI volumetric analyses were performed using FreeSurfer image analysis suite v6.0 [36]. All MRIs were read clinically by a neuroradiologist.

### Cortical diffusivity analysis

Cortical diffusivity analysis (CDA) was applied to MRI diffusion tensor imaging data. The method extracted a measure of cortical diffusion perpendicular to the axis of the mini-column structure in the cortical gray matter [37], which is distinct from axial or radial diffusivity or other diffusivity values. The analysis averages data from the principal diffusion direction across a cortical region. The diffusion perpendicular to the mini-column axis will tend to be high in cortex with greater cytoarchitectural disruption or cell loss. The concordances given here are based on mean whole brain values compared to our expectations of the typical thresholds associated with dementia or prodromal dementia; a categorization value of 1 indicates high values associated with high microstructural disruption and risk of dementia, while 0 indicates lower values, usually indicating lower risk.

### PET acquisition and analysis

All PET imaging was conducted with a Siemens mCT 40-slice 4RPET/CT camera (Siemens Healthcare) (see Supplementary Methods). Participants were injected with 370 MBq (10 mCi) of [^18^F]AV45. Image acquisition began ~60 min post-injection and lasted for 20 min. MIM Software Version 6.1 was used to measure standard uptake value ratio (SUVr) for [^18^F]AV45 PET analysis in six predefined anatomically relevant cortical regions of interest (frontal, temporal, parietal, precuneus, anterior cingulate, and posterior cingulate cortex); the whole cerebellum used as a reference region [38, 39]. Amyloid SUVr values ≥1.10 were classified as amyloid-positive.

[^18^F]AV1451 imaging was performed on day 2, after a 24-h washout period. Participants were injected with 370 MBq (10 mCi) of [^18^F]AV1451. Image acquisition began ~80 min post injection and lasted for 20 min [40, 41]. [^18^F]AV1451 images were read by a neuroradiologist and deemed “positive” if the subject exhibited ligand retention in areas not known for non-specific or off target binding. SUVr images were calculated as the uptake at each voxel divided by the mean uptake within the inferior cerebellar gray matter ROI, which was identified from the T1-MPRAGE using the spatially unbiased atlas template of the cerebellum and brainstem (SUIT) v3.2 [42] (see supplemental methods).

### Plasma and serum NfL quantification

Human and rat blood samples were collected and processed to isolate plasma (human) or serum (rats). Samples were stored at −80 °C, and shipped frozen to the Sahlgrenska Academy at the University of Gothenburg. NfL levels in plasma were measured on the ultrasensitive single molecule array (Simoa) platform (Quanterix, Lexington, MA, USA), a magnetic bead-based digital ELISA that allows detection of proteins at subfemtomolar concentrations, using an in-house assay as described [43]. All samples were analyzed using the same batch of reagents by certified laboratory technicians who were blinded to clinical information or experimental condition in animal studies.

### Statistical analysis

Statistical testing for animal studies employed unpaired *t* tests. For pairwise group comparisons of tau PET or serum NfL data, we used the nonparametric Mann–Whitney *U* test. For multiple group comparisons of serum NfL and tau PET, we used one-way ANOVA. The association between serum NfL and neuropsychological measures as well as SUVr uptake were tested using Spearman’s rank correlation. Statistical tests were performed using GraphPad Prism 7.0 (San Diego, CA), SPSS 25.0 (Chicago, IL), or R (v.3.0.3, The R Foundation for Statistical Computing).

## Results

### p-tau was increased in rat brains following blast exposure

**Fig. 1:**
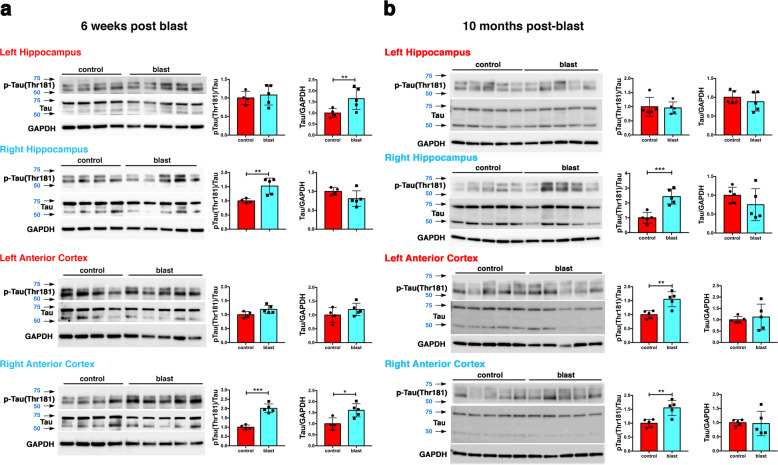
Hyperphosphorylated tau in hippocampus and anterior cortex of blast-exposed rats. Tau phosphorylation was analyzed at 6 weeks **a** and at 10 months **b** after blast exposure. Top row, AT270, middle row, total tau (tau), bottom row GAPDH. Graphs indicate p-tau levels expressed as the ratio of p-tau to total tau. Error bars indicate standard deviation (SD) (*p < 0.05, **p < 0.01, ***p < 0.001 vs. controls, unpaired *t*-tests). *n* = 5/group except for control at 6 weeks (*n* = 4). Size markers (kDa) are indicated by arrows next to each panel. p-tau blots were sequentially reprobed for total tau followed by GAPDH.

Rat tau consists of six isoforms containing 3 or 4 repeat domains [44]. On Western blots probed with the antibody AT270 directed against pThr181, rat p-tau shows two main bands between the 50 and 75 kDa molecular weight markers (Fig. 1). Western blot analysis with AT270 showed that 6 weeks following blast exposure, p-tau was increased in the right anterior cortex and right hippocampus but not in left anterior cortex and left hippocampus (Fig. 1a). Total tau was increased in right anterior cortex and left hippocampus. The increased tau phosphorylation at Thr181 in the anterior cortex was validated using two additional antibodies that recognize the pThr181 site. Both showed increased tau phosphorylation in right anterior cortex (Supplementary Fig. 1).

**Fig. 2:**
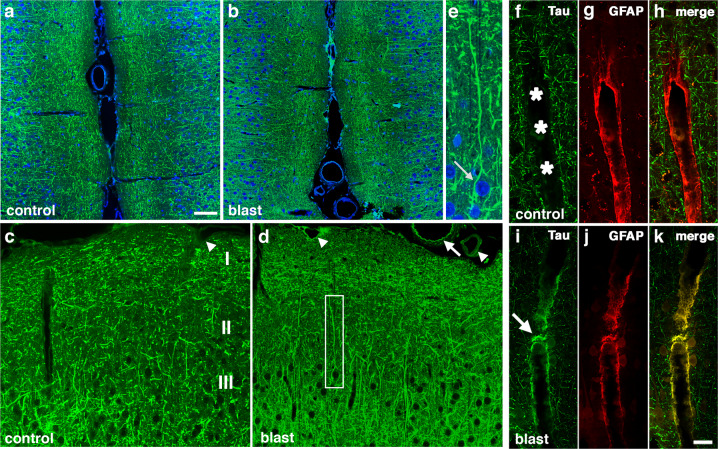
Immunostaining and distribution of p-tau in blast-exposed rat brain. **a**–**e** increased and somatodendritic redistribution of p-tau in the anterior cingulate cortex and motor cortex of blast-exposed rats 6 weeks following blast exposure. Shown are sections of the anterior cingulate **a**–**e** of control **a**, **c** and blast-exposed rats **b**, **d**, **e** immunostained with AT270 (green) counterstained with DAPI (blue). Cortical layers are indicated in panel **c**. Note the general increase of p-tau in all cortical layers in the blast-exposed animals **b**, **d**. **e** Higher power image of the neuron outlined by the white box in panel **d** showing prominent somatodendritic localization of p-tau. **f**–**k** perivascular p-tau in astroglial processes 10 months after blast-exposure. Penetrating cortical vessels from control **f**–**h** and blast-exposed rats **i**–**k** AT270 (green, **f** and **i**) and GFAP (red, **g** and **j**). The arrow in panel **i** indicates p-tau staining. An arrow in panel **d** indicates p-tau staining that appears to be in an elastic membrane. Arrowheads in panel **d** indicate other examples of perivascular tau staining. A penetrating cortical vessel that was not stained is indicated by an arrowhead in panel **c**. Scale bar: 50 μm **a**, **b**, 25 μm **c**, **d**, 10 μm **e**. Scale bar for panels **f**–**k**: 20 μm.

At 10 months after blast exposure, p-tau (Thr181) levels were increased in left and right anterior cortex (Fig. 1b) but not posterior cortex or the amygdala (Supplementary Fig. 2). In the hippocampus, p-tau levels were increased on the right side but not left (Fig. 1b). Total tau levels were unchanged in all regions examined (Fig. 2b and Supplementary Fig. 2). Increased tau phosphorylation at Thr181 was observed in the right anterior cortex and right hippocampus in another cohort of blast-exposed rats studied 12 months after blast exposure (Supplementary Fig. 3).

To determine whether tau phosphorylation was altered at additional sites, we used antibodies that recognize p-tau at Ser 202, Ser 202/Thr 205 (AT8), Thr 231, Ser 396, and Ser 404 (Supplementary Table 1) at 10 months post-blast exposure in cortex and hippocampus. Western blotting with CP13 (p-Ser 202) revealed increased p-tau in right anterior cortex (Supplementary Fig. 4). No changes in phosphorylation status were found at three other sites (Thr 231, Ser 396, and Ser 404) (Supplementary Figs. 5–7).

### Increased p-tau and somatodendritic redistribution of tau was detected in rat brain following blast exposure

Western blot analysis showed that p-tau was quantitatively increased in rat brains following blast exposure. To examine whether the pattern of p-tau distribution was altered following blast exposure we examined brain sections immunostained with AT270. As early as 6 weeks after blast, changes in p-tau immunostaining were evident in the anterior cingulate cortex of blast-exposed animals where many fine neuritic processes were labeled with AT270 (Fig. 2a–d). While dendritic staining was visible in control and blast-exposed sections, some neurons in blast-exposed animals contained perikaryal accumulations of p-tau (Fig. 2e, arrow) that were not observed in controls. In the anterior cortex at 10 months after blast exposure, while no perikaryal accumulations of tau were observed, many fine neuritic processes in cortical layers I–III of the primary motor and sensory/motor forelimb regions were labeled with AT270 (Supplemental Fig. 8), a pattern not observed in controls. As the anterior cortical samples used for Western blotting included all of the neocortex anterior to the optic chiasm, the immunostaining in Fig. 2 and Supplementary Fig. 8 can be considered the histological correlate of the anterior cortical samples studied in Fig. 1. Consistent with results from Western blots, abnormal p-tau accumulation was also observed in the hippocampus of blast-exposed rats (Supplementary Fig. 9).

### Phosphorylated-tau accumulated in perivascular astroglial processes

Figure 2f–k shows dual immunolabeling with AT270 and GFAP of penetrating cortical vessels from control and blast-exposed rats examined at 10 months following the last blast exposure. Compared to controls, blast-exposed rats exhibited perivascular labeling of vessels with AT270 (Fig. 2i), which co-localized with GFAP (Fig. 2k). Supplementary Figs. 10 and 11 show additional examples of large and small thalamic vessels from rats sacrificed 10 months after blast exposure that were cut in cross section and stained for p-tau and GFAP. P-tau-like immunoreactivity was present in perivascular GFAP-immunoreactive glial processes.

Abnormal perivascular tau accumulation was also visible in meningeal vessels lining the anterior cingulate cortex (Fig. 2d). This labeling sometimes had a pattern that suggested staining of the internal elastic membrane (arrow in Fig. 2d). However, dual immunostaining with AT270 and α-smooth muscle actin (αSMA, Supplementary Fig. 12), showed that the p-tau immunoreactivity did not co-localize with αSMA staining and was in elements outside the smooth muscle layer although in some more severely affected vessels like the one in the molecular layer of the hippocampus illustrated in Supplementary Fig. 10g–i, p-tau-like immunoreactivity was visible not only in astrocytic but possibly other vascular elements.

#### MRI and cortical diffusivity analysis results

Brain MRI results from five veterans were normal, while those from the remaining subjects displayed minimal nonspecific foci of T2/FLAIR hyperintensity (Table 1). Quantitative volumetric assessment of various regions of interest on both left and right hemispheres (such as hippocampus, cerebellum, caudate, putamen, and ventricles) did not reveal consistent differences between veterans and the comparison group. Cortical diffusivity analysis identified 1 of 7 members of the comparison group and 5 of 9 veterans with a score of 1 (high microstructural disruption).

#### Molecular imaging results

**Fig. 3:**
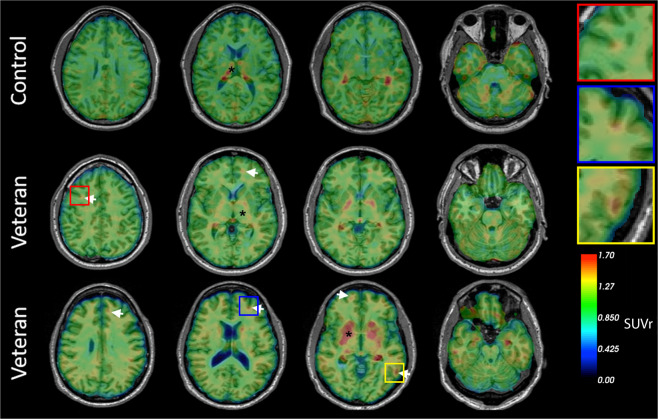
Representative transaxial brain images of [^18^F]AV1451 PET of veterans with history of multiple blast exposures. Veterans 1 and 2 show cortical ligand retention at the white/gray matter junction, as is characteristic of the distribution of tauopathy in CTE (white arrows). * Indicates areas of non-specific binding and uptake. The top row represents a cognitively healthy control. Insets show higher magnification of foci of [^18^F]AV1451 ligand retention.

Using [^18^F]AV45, all veterans and members of the comparison group were identified as amyloid-negative as all ROIs SUVrs were below the cutoff level of 1.1. With regard to interpretation of [^18^F]AV1451 data, qualitative reads by a neuroradiologist (LK) led to identification of excess ligand retention in the brains of 5 of 10 veterans (Fig. 3). While the amount of ligand retention varied, the patterns of retention consistently resembled the pathognomonic distribution of CTE tauopathy, with foci of [^18^F]AV1451 retention located deep in the sulci at the white/gray matter junctions in frontal, parietal, and occipital brain regions (see inset Fig. 3). No retention of [^18^F]AV1451 was observed in the comparison group with the exception of regions previously reported to show nonspecific or off-target binding [44, 45]. Overall, [^18^F]AV1451 retention was highest among veterans with the most severe clinical symptoms as determined from self-reported medical history, neurological assessment, and neuropsychological tests (Table 1). The brains of veterans with milder postconcussive symptoms not suspect for CTE retained little or no detectable [^18^F]AV1451.

### NfL levels in blood

We observed no difference in plasma NfL concentrations when all veterans were compared to the comparison group. In addition, there was no correlation between plasma NfL levels and PET SUVr values in six ROIs when all groups were pooled (Supplementary Fig. 13). However, when we examined plasma NfL following stratification by tau-positivity (i.e., retainers of excess [^18^F]AV1451) vs. tau-negative veterans), we observed that the highest NfL levels were among veterans with excess [^18^F]AV1451 ligand retention (Mann–Whitney test, *p* = 0.031; Supplementary Fig. 14a). Interestingly, veterans with high cortical diffusivity analysis scores had higher plasma NfL levels as well as [^18^F]AV1451 ligand retention. Correlations among NfL levels, age, and various neuropsychiatric measures revealed no obvious relationships (Supplementary Fig. 14).

We also measured NfL levels in sera collected from rats at 4 or 10 months following blast exposure. We did not find differences between blast-exposed and controls at 10 months after blast exposure (Supplementary Fig. 15). At 4 months after blast exposure, there was a stratification of the blast-exposed into rats with high and low NfL levels with 5 out of 10 blast-exposed animals having serum levels >100 pg/mL compared to only 1 out of 6 controls (Supplementary Fig. 15). Means at 4 months were different if one outlier was removed from the control group (*p* < 0.02, Mann–Whitney test). Future studies will be needed to determine whether there is any correlation in individual animals between serum NfL levels and the degree of CNS tauopathy.

### Clinical and neuropsychological assessment

The age-matched comparison group and veteran groups were significantly different in IQ, the comparison group having a higher average IQ (Table 2). The groups did not differ significantly on most tests of attention or processing speed; similarly, they did not differ on visual or verbal memory. However, the veteran group had significantly lower fine motor dexterity and reported significantly higher levels of depression. The groups did not differ on self-reported alcohol use (Table 2).

## Discussion

In this study, we identified biomarker signatures in human veterans exposed to IED blasts in theater and rats exposed to repetitive low-level blasts in a shock tube. The veterans all reported histories of blast exposure and had chronic behavioral and cognitive complaints. The number of human blast exposures varied from as few as 1 to as many as 50 or more but were all associated with at least 1 mTBI by clinical history. The rat model was developed to mimic a level of blast exposure that would be associated with human mTBI or a subclinical blast exposure [34]. Rats exposed to this blast protocol exhibit a range of chronic anxiety and PTSD-like behavioral traits [26, 29, 31] and PTSD-related symptoms were prominent in the clinical presentations of the human veterans. Rats received three blast exposures delivered one per day on 3 consecutive days. While not exactly paralleling the human exposure history, the rats model the human condition in exhibiting a chronic neurobehavioral phenotype that resulted from a blast exposure chosen to mimic a human mTBI or subclinical blast exposure.

The primary findings of this study reveal that: (1) repeated mTBI induced by blast-overpressure increased levels of p-tau-like immunoreactivity in the brains of rats from 6 weeks to 12 months following injury, with elevations observed in anterior cortex and hippocampus. Immunohistochemical staining revealed increased p-tau and somatodendritic redistribution during the same time frame. (2) Repeated mTBI induced by blast overpressure produced abnormal perivascular p-tau accumulation in astroglial processes surrounding blood vessels in rats. (3) 50% of veterans with a history of multiple blast exposure showed excess cortical retention of [^18^F]AV1451 at the white/gray matter junctions, matching the anatomical distribution of tauopathy previously observed in postmortem studies of subjects with CTE [18]. (4) Finally, circulating plasma NfL levels were highest among veterans who exhibited [^18^F]AV1451 retention.

Astrocytic tau pathology including p-tau accumulation is a prominent feature of a variety of human neurodegenerative diseases particularly certain forms of frontotemporal lobar degeneration [47]. In CTE aggregates of p-tau are found in neurons and astroglia in perivascular locations especially within the depths of the superficial cortical sulci [18]. In our well-established and characterized battlefield-relevant rat model of blast-induced mTBI, we found that animals exhibited increased levels of immunoreactive p-tau following blast exposure in the anterior cingulate and anterior motor/sensory cortices. Prior studies have addressed tau processing in experimental animal models of blast TBI, with many reporting elevated levels of tau in brain or blood [21, 46–65]. Two studies reported the presence of tau oligomers following blast exposure [59, 61] and others have suggested that p-tau metabolism following blast may be regulated by *APOE* genotype [62]. P-tau-like accumulations have also been observed in mouse retinal neurons and glia following blast injury [66], and in the superficial layers of the cerebral cortex [21]. Meabon et al. [60] also described perivascular accumulations of tau in a mouse model of blast injury. To our knowledge, our data are the first to describe hyperphosphorylated tau-like immunoreactivty in the brain for up to 12 months following blast exposure in a rat model known to produce a chronic anxiety-like behavioral phenotype. Thus, this model may be a suitable platform for studying the chronic neuropathology of repeated blast mTBI and its relationship to behavioral changes.

The changes in the anterior but not posterior cortex are of potential relevance to the behavioral phenotype. Current models of PTSD suggest that frontal structures—in particular the prefrontal cortex —are involved in the development of PTSD [67, 68]. Inadequate frontal inhibition of the amygdala is suggested to form the basis for heightened responses to psychological threats [67]. We have observed increases in the microtubule-associated protein stathmin 1 in the amygdala of blast-exposed animals months after exposure that is associated with increased anxiety [26]. Further study will be required to determine whether accumulation of p-tau species in the anterior cortex suggests preferential effects of blast on frontal regions that ultimately lead to disinhibition of the amygdala and increased anxiety.

An aspect of the pattern of p-tau accumulation that remains puzzling is the laterality of the effect. If a structure was affected the right side was always involved with the corresponding left side never affected unless the right was as well, an effect that has been reproducible across multiple cohorts of animals. The blast exposure is delivered as a straight frontal exposure and there should be no systematic variation in the rat’s placement within the blast tube that would cause the right hemisphere to be differentially impacted. We can only speculate that this pattern reflects some laterality of hemispheric function in the rat, which causes the right side to respond differently to the shock wave or is a feature of the evolution of the injury. Indeed, although not as well appreciated as in humans, rats and mice have been known to exhibit paw preference and hemispheric laterality for complex behavioral functions since the 1970s [69]. Hemispheric dominance has been in particular found to affect spatial memory in rodents [70, 71] and behavioral asymmetries have been correlated with biochemical asymmetries in rodent brain, particularly in the dopaminergic system [72]. Tau is known to affect the stress response in mice [73] and hemispheric asymmetries have been found in expression of brain-derived neurotrophic factor and neurotrophic tyrosine kinase receptor type 3 in stress-resilient rats [74]. Whether asymmetries in p-tau expression could also be part of a lateralized stress response is unknown. Future studies will be needed to explore in particular the pattern of evolution of tau pathology in rat brain following blast injury.

Our human studies provide antemortem evidence of tauopathy in half of symptomatic veterans using PET imaging. We have previously demonstrated [^18^F]AV1451 retention in retired NFL players [39, 75]. The anatomical distribution of the retained ligand matches that previously described in other in vivo studies as well as postmortem histopathological studies in veterans and athletes [20–22]. The location of the tau ligand at the white/gray matter junction corresponds to reports of diffusion imaging and axonopathy resulting from blast trauma and with CTE [24, 76, 77]. Recently, Chen et al. [78] used the FDDNP tau ligand to demonstrate excess ligand binding in military personnel with histories of mTBI similar to retired athletes with mTBI, with localization of that ligand retention in the amygdala, midbrain, thalamus, and pons, as well as frontal and anterior and posterior cingulate regions [78]. In another study, 16 veterans underwent PET imaging with [^18^F]AV1451, and excess ligand retention was observed in the cerebellar, occipital, inferior temporal, and frontal regions [79]. While tau ligand uptake was observed in military personnel with mTBIs, the regional localization of the ligand differed from that reported here (crowns of the gyri in ref. [71] vs. depths of the sulci in our study). What should also be noted is that [^18^F]AV1451 and FDDNP show different binding patterns and what is considered significant signal with one ligand (such as the thalamus and pons with FDDNP) originates from regions that cannot be reliably assessed with [^18^F]AV1451 because these regions (thalamus, pons) are among those confounded by known patterns of non-specific or off-target retention of [^18^F]AV1451 [45, 46]. Moreover, methods for the quantification and interpretation of the retention of various PET ligands are not yet standardized. In studies of AD, the cerebellum is commonly used as a reference region since there is little to no tau pathology found. In regard to TBI and other tauopathies, the region of choice for normalization varies across studies. For example, some studies use the entire cerebellum [78]; others have used the inferior cerebellum [80, 81]; and others the isthmus cingulate [79]. However, in TBI, certain parts of the cerebellum have been known to be affected in some cases and ligand retention observed [79]. Further refinement of the methodology and analysis of tau PET imaging will be needed for its efficient application to TBI.

With regard to cortical diffusivity analysis values and changes in the microstructure of white and gray matter, it is known that the accumulation of tau pathology (specifically, NFT density) is correlated with disrupted cellular organization, including disruption of the columnar arrangement of cells, dendrites, and axon bundles in the cerebral cortex [37]. One possible interpretation is that the microgeometry in cortical gray matter is sensitive to microanatomical disruption due to mTBI associated with elevated tau levels. It is uncertain which aspect of altered cellular structure is particularly vulnerable, but the change identified here indicates sensitivity in the direction perpendicular to the mini-column structure. This may implicate interlaminar dendrite and axon bundles. The alteration in this particular aspect of cortical diffusion could prove to be a useful biomarker.

While these results are highly promising for developing a clinical biomarker signature that may one day support the diagnosis of CTE during life, there are limitations. The development of in vivo diagnostics of CTE is in its infancy and replicating these findings in larger cohorts with a range of TBI severity and frequency is warranted. Sample size in the current study precluded more detailed statistical analyses; however, the trends pointed toward higher plasma NfL levels in veterans who retained excess [^18^F]AV1451. Moreover, the use of [^18^F]AV1451 for the in vivo detection of tauopathy in living veterans must be validated with postmortem neuropathological assessments. The pilot data in rats is also suggestive of an NfL elevation in serum at 4 months after blast exposure. While the present studies by themselves may not be conclusive they support an emerging consensus that NfL and other biomarkers may play some role in monitoring post concussive recovery and warrant further study in humans and in animal models.

Finally, we recognize that the healthy subject comparison group was made up of civilians who had no military exposure. The ideal control group would have been a group of non-blast exposed deployed veterans as well as veterans with no history of any TBI. However, we were not able to recruit such individuals at the time of this study. There are other studies that have performed imaging in veterans that have used as “healthy controls” civilian populations rather than non-blast or non-TBI military controls [79, 82] or have made comparisons to athletes and civilians [83].

Definitive studies will be required to establish both qualitative and quantitative associations between biomarker changes and chronic behavioral effects of blast. Anecdotal reports using other tauopathy ligands are also promising, and various ligands must be compared in the same patient at the same time in order to compare and contrast how these ligands perform [78, 84]. These findings have implications for understanding the relationship of chronic blast-related injury to human neurodegenerative diseases including CTE. The current data provide evidence, in two species, to support the existence of a relationship involving blast injury, and clinical neuropsychiatric syndromes. Tauopathy, was present in the animal model and in a subgroup of the veterans.

## Supplementary information

Supplementary Information

Supplementary Figure 1

Supplementary Figure 2

Supplementary Figure 3

Supplementary Figure 4

Supplementary Figure 5

Supplementary Figure 6

Supplementary Figure 7

Supplementary Figure 8

Supplementary Figure 9

Supplementary Figure 10

Supplementary Figure 11

Supplementary Figure 12

Supplementary Figure 13

Supplementary Figure 14

Supplementary Figure 15
