# Supplementary material for: Brain and blood biomarkers of tauopathy and neuronal injury in humans and rats with neurobehavioral syndromes following blast exposure
Source: Mol Psychiatry. Author manuscript; Available in PMC 2021 Aug 25. (PMC7484380; doi:10.1038/s41380-020-0674-z)
Supplement: Supplementary file 1 [file NIHMS1615402-supplement-Supplementary_Information.docx]

**Supplementary Information**

**Brain and blood biomarkers of tauopathy and neuronal injury in humans and rats with neurobehavioral syndromes following blast exposure**

Dara L. Dickstein, Rita De Gasperi, Miguel A. Gama Sosa, Georgina Perez-Garcia, Jennifer A. Short, Heidi Sosa, Gissel M. Perez, Anna E. Tschiffely, Kristen Dams-O’Connor, Mariel Y. Pullman, Karin Knesaurek, Andrew Knutsen, Dzung L. Pham, Lale Soleimani, Barry D. Jordan, Wayne A. Gordon, Bradley N. Delman, Gleb Shumyatsky, Pashtun-Poh Shahim, Steven T. DeKosky, James R. Stone, Elaine Peskind, Kaj Blennow, Henrik Zetterberg, Steven A. Chance, Mario Torso, Lale Kostakoglu, Mary Sano, Patrick R. Hof, Stephen T. Ahlers, Sam Gandy and Gregory A. Elder 

**Supplementary Information:** Supplementary data, figures, and tables are available at Molecular Psychiatry’s website. 

**Supplementary Methods**

**Regional brain dissection**

To dissect the various brain regions the cerebellum was removed and the brain was placed ventral side up and coronal cuts were made through the optic chiasm and anterior commissure. The cortical tissue surrounding this piece of tissue was defined as the anterior cortex. To obtain the amygdala fraction, the tissue lateral to the hypothalamus between its caudal and rostral border and ventral to the rhinal sulcus on either side was dissected. After dissection of the amygdala the tissue was turned dorsal side up and the hippocampus dissected based on its typical morphology after the cerebral hemispheres were reflected out. The posterior cortex included the remainder of the cerebral tissue after the removal of the caudate putamen.

**Western Blot analysis**

Tissue was homogenized in 0.1 M Tris HCl buffer pH 7.4, containing 0.15 M NaCl, 5 mM EDTA, 1% Triton X100/0.1% SDS and a protease and phosphatase inhibitor cocktail (Halt, Pierce, Rockford IL, USA). Protein concentration was determined with the BCA reagent (Pierce).  Protein samples (50 μg) were separated by SDS-PAGE and blotted onto polyvinylidene difluoride (PVDF) membranes (Millipore Corporation, Billerica, MA, USA). Blots were blocked with 50 mM Tris HCl, pH 7.6, 0.15 M NaCl, 0.1% Tween-20 (TBST), 5% nonfat dry milk and probed overnight with the relevant primary antibody diluted in blocking solution. Blots were then incubated for 1.5 h with the appropriate horseradish peroxidase (HRP) conjugated secondary antibody (GE Healthcare Lifesciences, Piscataway, NJ, USA) diluted in blocking solution (1:7500) and bands were visualized by ECL Prime (GE Healthcare Lifesciences) and imaged with Imager 600 imaging station or by exposure to HyBlot CL film (Denville Scientific). The primary antibodies utilized are indicated in Supplementary Table 1. All p-tau blots were sequentially reprobed to determine total  tau and GAPDH levels. For reprobing, the membranes were stripped with Re-Blot Plus strong stripping solution (Millipore) or ReblotPlus western blot stripping buffer (ThermoFisher) according to the manufacturer’s instructions. Quantification was performed using Image Quant TL software (GE Healthcare Lifesciences). 

**MRI and PET acquisition and analysis**

The MRI T1-weighted images were obtained on a 3-Tesla Siemens Biograph MR system (TR = 2300 ms, TE = 2.98 ms, 176 sagittal slices, 1.2 mm slice thickness, voxel-size of 1 x 1 x 1.2 mm, flip angle = 90°). All scans were performed at the ISMMS Hess Center for Science and Medicine. MRI volumetric analyses was performed using FreeSurfer image analysis suite v6.0^36^; ([http://surfer.nmr.mgh.harvard.edu/](http://surfer.nmr.mgh.harvard.edu/)) with skull-stripping performed using the Multi-cONtrast brain STRipping method (MONSTR; https://www.nitrc.org/projects/monstr/). All MRIs were evaluated by a neuroradiologist and were read clinically.

All PET images were acquired in three dimensions, using a one-frame and one-bed position TOF Dynamic All PASS filter. Reconstructions were performed utilizing iterative reconstruction, with 24 subsets and 4 iterations. The z-axis filter was standard, summed images 5.0 mm full-width/half-maximum Gaussian filter was used. The field of view was 50 cm in diameter, with 74 total slices of 3 mm thickness. A CT scan was obtained for attenuation correction. 

The [^18^F]AV1451 images were rigidly registered to the T1-MPRAGE using the rigid registration algorithm in the Advanced Normalization Tools (ANTs) v2.1^75^. Voxels within the inferior cerebellar grey matter were identified using the spatially unbiased atlas template of the cerebellum and brainstem (SUIT) v3.2^42^ (http://www.diedrichsenlab.org/imaging/suit.htm) and the cerebellar grey matter label from FreeSurfer (aparc+aseg.mgz)^36^. Specifically, SUIT was used to segment the cerebellum and brainstem, and the SUIT template was registered to the MPRAGE using the nonlinear registration algorithm in the ANTs toolkit. The inferior cerebellar ROI (indices 6, 8-28, 33, and 34) was then transformed to the subject space using nearest neighbor interpolation. The inferior cerebellar grey matter ROI was defined as voxels labeled both as cerebellar grey matter via FreeSurfer and inferior cerebellum via SUIT. SUVr images were calculated as the uptake at each voxel divided by the mean uptake within the inferior cerebellar grey matter ROI. For the analysis, average SUVr values were computed in the hippocampus, caudate nucleus, thalamus, putamen, amygdala, precuneus, anterior cingulate cortex, posterior cingulate cortex, and pallidum, as well as the frontal, parietal, and temporal lobes. 

**Supplementary Figure Legends**

**Supplementary Figure 1**

Validation of increased tau phosphorylation at Thr181 in the anterior cortex in rats 6 weeks following the last blast exposure (n=4 for controls and 5 for blast-exposed). The blots were probed using a rabbit monoclonal antibody from Cell Signaling (a) or a rabbit oligoclonal antibody from ThermoFisher (b) both recognizing pThr181-tau. Levels of p-tau are expressed as the ratio of p-tau to total tau. Error bars indicate SD (*p<0.05, **p<0.01 vs. controls, unpaired t-tests). As shown in Fig.1b increased tau phosphorylation at Thr181 was detected in the right anterior cortex only. Size markers (kD) are indicated by arrows next to each panel. p-tau blots were reprobed for total tau.

**Supplementary Figure 2** 

Tau phosphorylation in the posterior cortex and amygdala at 10 months following the last blast exposure (n=5 per group). Blotting was performed with the AT270 antibody as described in Fig. 1. Levels of p-tau are expressed as the ratio of p-tau to total tau. No difference in pThr181-tau or total tau levels was found in these regions.  Size markers (kD) are indicated by arrows next to each panel. p-tau blots were sequentially reprobed for total tau followed by GAPDH.

**Supplementary Figure 3** 

Tau phosphorylation in the hippocampus and anterior cortex in rats 12 months following the last blast exposure (n=8 for control and 9 for blast-exposed). Blotting was performed with AT270 antibody (pThr181) as described in Fig. 1. Levels of p-tau are expressed as the ratio of p-tau to total tau. Total tau levels were normalized to GAPDH. Tau phosphorylation at Thr181 was elevated in the right hippocampus and right anterior cortex following blast injury. A decrease in total tau was observed the left hippocampus and p-tau was decreased in the left anterior cortex. Error bars indicate SD (* p< 0.05, **p<0.01 vs. controls, unpaired t-tests). Size markers (kD) are indicated by arrows next to each panel. p-tau blots were sequentially reprobed for total tau followed by GAPDH.

**Supplementary Figure 4**

Tau phosphorylation in the anterior cortex in rats 10 months following the last blast exposure (n=5 per group). Blotting was performed with the p-tau antibody CP13 (pSer202) as described in Fig. 1. Levels of p-tau are expressed as the ratio of p-tau to total tau. Error bars indicate SD (**p<0.01 vs. controls, unpaired t-tests). Size markers (kD) are indicated by arrows next to each panel. p-tau blots were reprobed for total tau.

**Supplementary Figure 5**

Tau phosphorylation in the anterior cortex in rats 10 months following the last blast exposure (n= 5 per group). Blotting was performed with a rabbit oligoclonal antibody to pThr231 as described in Fig. 1. Levels of p-tau are expressed as the ratio of p-tau to total tau. Size markers (kD) are indicated by arrows next to each panel. p-tau blots were reprobed for total tau.

**Supplementary  Figure 6**

Tau phosphorylation in the anterior cortex in rats 10 months following the last blast exposure (n=5 per group). Blotting was performed with the mouse monoclonal antibody PHF13 as described in Fig. 1. Levels of p-tau are expressed as the ratio of p-tau to total tau. Size markers (kD) are indicated by arrows next to each panel. p-tau blots were reprobed for total tau.

**Supplementary Figure 7**

Tau phosphorylation in the anterior cortex in rats 10 months following the last blast exposure (n=5 per group). Blotting was performed with an antibody against pSer404 as described in Fig. 1. Levels of p-tau are expressed as the ratio of p-tau to total tau. Size markers (kD) are indicated by arrows next to each panel. p-tau blots were reprobed for total tau.

**Supplementary Figure 8**

Increased p-tau immunostaining in the anterior cortex of blast-exposed rats at 10 months post-blast. Sections from the anterior cortex including the primary motor and sensory/motor forelimb regions of blast exposed (a, b, c) and control (d, e, f) were immunostained with the anti-phospho tau antibody A270 (a, c, d, f) and counterstained with a DAPI nuclear stain (b, e). Rat cortical layers I-III are indicated in panel (b). Note the increased A270 staining in layers I to III indicated by arrows in the blast-exposed animal. Panels (c) and (f) show higher power images of the staining in layers I and II. Scale bar: 100 μm for a, b, d and e; 50 μm for c and f.  

**Supplementary Figure 9**

AT270 immunostaining in the hippocampus of a control **(**a**)** and blast-exposed rat **(**b**)** 10 months following the last blast exposure. Note the increased immunostaining in the hilus (asterisks) of the dentate gyrus (DG) in the blast-exposed animal. Scale bar: 75 μm.

**Supplementary Figure 10.** 

Perivascular p-tau in astroglial processes. Panels (a-f**)** show thalamic vessels from control (a-c) and blast-exposed rats (d-f) sacrificed 10 months after the last blast exposure immunostained with AT270 (green, a and d) and GFAP (red, b and e). Merged images are shown in panels **(**c) and (f**)**. Arrows in panel (f**)** indicate p-tau staining in two smaller vessels. Scale bar for panels (a-f**)** is 25 μm. In panels **(**g-i**)** perivascular tau staining with AT270 is shown in a blood vessel in the molecular layer of the hippocampus of a blast-exposed rat sacrificed 10 months after the last blast exposure. A vessel containing tau in GFAP immunostained processes and possibly other vascular elements is indicated by an arrow. An adjacent unaffected vessel is indicated by an arrowhead. Scale bar for panels (g-i) is 25 μm. 

**Supplementary Figure 11.** 

 P-tau in perivascular astroglial processes. Shown is a thalamic vessel from a blast-exposed rat sacrificed 10 months after the last blast exposure immunostained with AT270 (green, a and d) and GFAP (red, b and e). Merged images are shown in panels **(**c) and (f**)**. DAPI is shown in blue. Panels (d)-(f) show higher power images. Note the localization of p-tau immunostaining mostly within GFAP immunostained perivascular astroglial processes. Asterisks in panel (f**)** indicate endothelial cell nuclei identifiable by their elongated appearance. Scale bar: 40 μm for (a)-(c); 20 μm for (d)-(f). 

**Supplementary Figure 12.** 

Lack of p-tau in the vascular smooth muscle layer. Shown is a pial cortical vessel (a-d) or a thalamic vessel (e-h) from a blast-exposed rat sacrificed 10 months after the last blast exposure. Sections were immunostained with AT270 (green, a and e) and αSMA (red, b and f). DAPI is shown in blue. Merged images are shown in panels **(**c) and (g**)**. Panels (d) and (h) show higher power images of the boxed areas indicated in panels (c) and (g). Note the lack of co-localization of p-tau immunostaining with the αSMA stained smooth muscle layer. Scale bar: 40 μm for (a)-(c) and (e)-(g); 10 μm for (d) and (h). 

**Supplementary Figure 13.** 

Relationship between plasma NfL values and PET SUVr values in six ROIs in the healthy comparison group (controls), tau +ve and tau -ve veterans. Spearman rank correlations (R^2^) and p values are indicated.

**Supplementary Figure 14.** 

Relationship between plasma NfL in veterans and [^18^F]AV1451 positivity, age, MoCA and BDI-II  scores. In panel (a) between-group comparisons are shown for plasma NfL levels within subjects in the comparison group (control) as well as tau +ve and tau -ve veterans.   * indicates p = 0.031, Mann Whitney test. Panels (b)-(d) show correlations between plasma NfL in veterans and age (b), MoCA (c) and BDI-II (d) scores. Spearman rank correlations (R^2^) and p values are indicated. 

**Supplementary Figure 15.** 

Serum NfL levels in rats exposed to blast. Serum NfL was measured in rats at 4 months (n = 10 blast exposed and 6 controls) or 10 months (n = 5/group) after blast exposure. Data is presented as mean ± SEM. There were no differences between blast-exposed and control rats at either time point when all values were included (unpaired *t*-tests). Groups differed if the outlier in the control group (identified by the ROUT test) was eliminated and comparisons were made using a non-parametric Mann-Whitney test (p < 0.02).

| **Supplementary Table 1. Antibodies used for Western blot and immunohistochemistry** |  |  |  |  |  |  |  |
| --- | --- | --- | --- | --- | --- | --- | --- |
| ***Antibody*** | ***Specificity*** | ***Source*** | ***type*** | ***WB*** | ***IHC*** | ***Remarks*** |  |
| *AT270* | *Thr181* | *ThermoFisher* *MN1050* | *Mouse monoclonal* | *1:600* | *1:400* |  |  |
| *Phospho-Tau (Thr181)* | *Thr181* | *Cell Signaling* *12885* | *Rabbit monoclonal* | *1:1200* |  |  |  |
| *Phospho-Tau (Thr181)* | *Thr181* | *ThermoFisher* *710561* | *Rabbit oligoclonal* | *1:1000* | *1:400* |  |  |
| *Phospho-Tau (Ser404)* | *Ser404* | *ThermoFisher* *44-758G* | *Rabbit polyclonal* | *1:1000* |  | *No difference* |  |
| *PHF13* | *Ser396* | *Cell Signaling* *9632* | *Mouse monoclonal* | *1:1000* |  | *No difference* |  |
| *Phospho-Tau (Thr231)* | *Thr231* | *ThermoFisher* *710126* | *Rabbit oligoclonal* | *1:1000* |  | *No difference* |  |
| *CP13* | *Ser202* | *Dr Peter Davies* | *Mouse monoclonal* | *1:500* |  |  |  |
| *AT8* | *Ser202/Thr205* | *ThermoFisher* *MN1020* | *Mouse monoclonal* | *1:600* |  | *Very poor signal in WB* |  |
| *Tau* | *Total tau* | *Proteintech* *10274-1-AP* | *Rabbit polyclonal* | *1:600* |  |  |  |
| *GT239* | *GAPDH* | *GeneTex GTX627408* | *Mouse monoclonal* | *1:4000* |  |  |  |
| *GFAP* | *GFAP* | *Dr Virginia Lee* | *Rat monoclonal* | *1:500* |  |  |  |
| *α**SMA* | *α**SMA* | *Proteintech* *143-95-1-AP* | *Rabbit polyclonal* |  | *1:1,000* |  |  |
